# An Uncommon Case of Peripheral Osteoma of the Maxilla: A Case Report

**DOI:** 10.7759/cureus.33437

**Published:** 2023-01-06

**Authors:** Akanksha Shiradhonkar, Motilal Jangid, Vino Tito V Kurien, Ruchita Humne, Saleem Khan

**Affiliations:** 1 Periodontology, Saraswati Dhanwantari Dental College and Hospital, Parbhani, IND

**Keywords:** peripheral type, maxilla, electrocautery, benign, osteoma

## Abstract

Facial bone osteomas are uncommon, with only a few cases reported in the literature. Osteomas are benign neoplasms that are composed of well-differentiated, mature bones. There are three types of osteomas: central osteomas that develop from the endosteum, peripheral osteomas (PO) that develop from the periosteum, and extra-skeletal soft tissue osteomas that develop from the muscle. Both central and peripheral osteomas of the facial bones have been described. Peripheral osteomas have been reported in the frontal, ethmoid, and maxillary sinuses, but they are uncommon in the jawbone. It occurs at a younger age but is most prevalent in the sixth decade, with a female-to-male ratio of 1:2. The purpose of this case report was to examine the clinical manifestation and management protocol of such lesions using electrocautery.

## Introduction

An osteoma is a benign osteogenic tumour that originates from the emergence of the cancellous or compact "ivory" in the endosteum, periosteal osteoma, or extra-skeletal soft tissue osteoma, which typically appears in the muscle. The majority of peripheral osteomas (PO) appear to grow extremely slowly, exhibit swelling and asymmetry, and have no symptoms. Uncertainty surrounds its pathophysiology; some consider it to be a legitimate tumour, while others categorize it as a developmental aberration [1].

Osteomas are benign tumours of the paranasal sinuses that emerge quite commonly. They may be the result of a post-inflammatory or post-traumatic phase, according to some theories. One potential root cause is the activation of embryologic cartilaginous rests. Osteomas can have one or many nodules. When abundant, they are frequently linked to Gardner syndrome, which is characterized by several intestinal polyps with the potential to become cancerous, unerupted normal and supernumerary teeth, cysts, and skin fibromas.

Although peripheral osteomas are typically asymptomatic, they can induce oedema and asymmetry. Clinically, it has a confined appearance, is typically spherical and protuberant, and exhibits very slow continuous growth. Peripheral (mature) osteoma, also known as ivory osteoma, should be investigated as a differential diagnosis if any circumferential, single, bony, stiff, gradually growing, painless swelling that affects the oral cavity is seen [1]. Diverse pathologic entities, comprising exostosis, chronic localized sclerosing osteomyelitis, ossifying fibroma, chondroma, osteoblastoma, osteoid osteoma, osteosarcoma, and odontoma, should be included in the differential diagnosis [2].

"These lesions can occur at any age, but they have a marked preference for elderly persons and are twice as common in men as they are in women [3]."

Osteoma is treated by completely removing it surgically from the base, where it contacts the cortical bone. A substantial cosmetic deformity, a limitation in function, a significant growth rate, or a conclusive histopathologic diagnosis are all indications for surgical therapy. Before selecting a treatment regimen, it is important to always keep in mind that osteoma and Gardner's syndrome are closely linked. In conclusion, hard palate peripheral osteomas are uncommon bone lesions. When medical intervention is necessary, surgical excision is the preferred method [2].

## Case presentation

**Figure 1:**
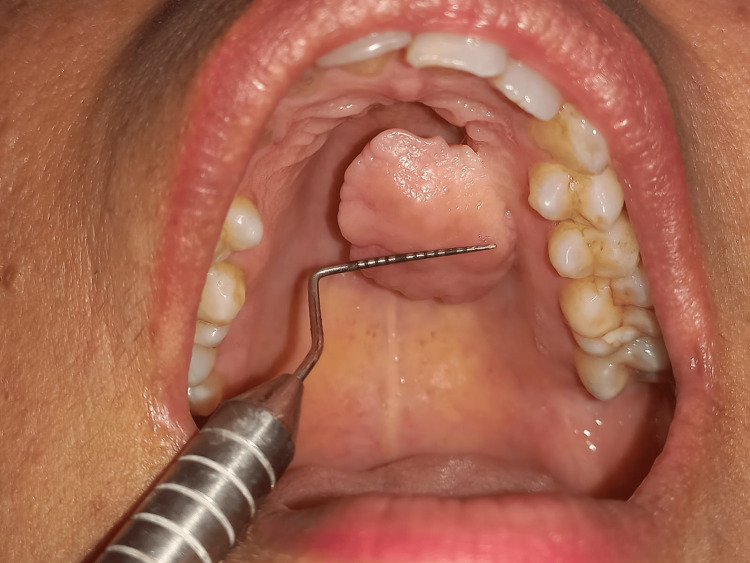
Pre-operative

A 42-year-old female patient reported to the Department of Periodontics with the chief complaint of swelling in the upper left back tooth region of the jaw for 15 years. She observed an asymptomatic peanut-sized swelling (Figure 1) in the palatal region, which has been slowly increasing in size. On clinical examination, the oral cavity showed a bony, hard, painless pedunculated mass of 17 mm × 20 mm attached to the palatal aspects of 23, 24, and 25. The overlying mucosa was intact with no ulcerations.

**Figure 2:**
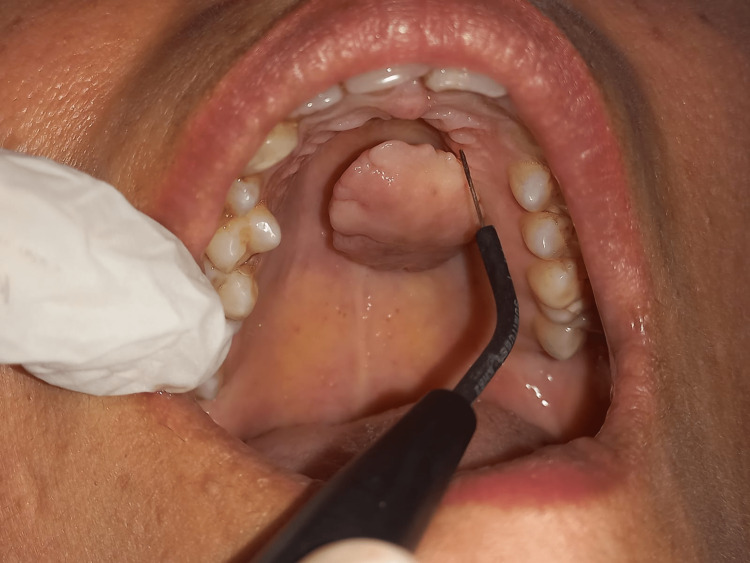
Excision using electrocautery

On systemic examination, the patient reported a history of hypertension for three years and was on medication for the same. There was no history of trauma. The patient did not report any deleterious habits. The treatment procedure was explained to the patient, and informed consent was obtained for the same. The patient underwent oral prophylaxis and was instructed to perform and maintain oral hygiene practices. A haematological investigation was performed. Electrocautery was employed for excision in the cutting and coagulation modes under local anaesthesia. Excision was executed using a single wire electrode (Figure 2), whereas coagulation was achieved using a ball electrode. Light brushstrokes were employed, and the point was consistently moved. In order to prevent heat buildup and potential tissue destruction, prolonged and repetitive electrode stimulation of the tissue was avoided.

**Figure 3:**
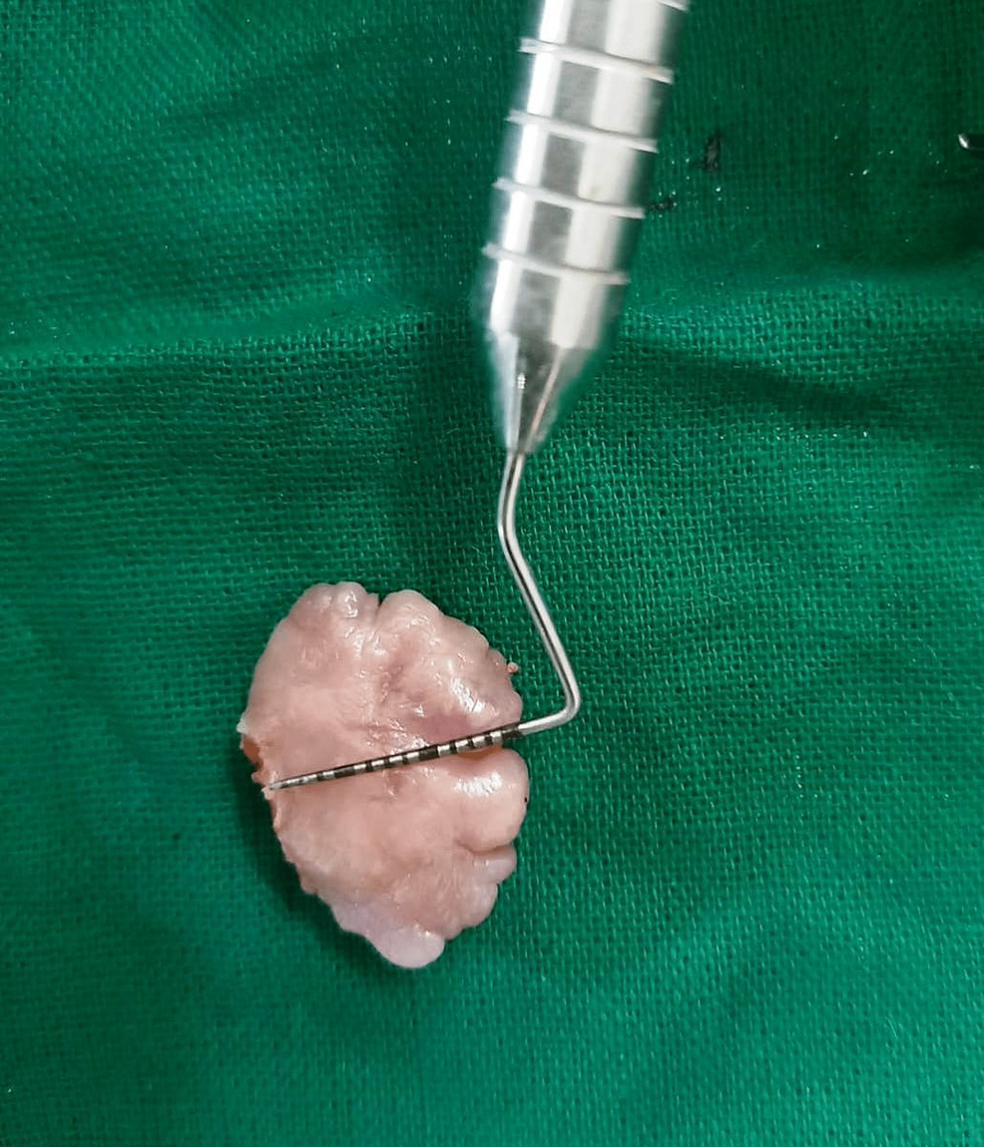
Excised tissue

**Figure 4:**
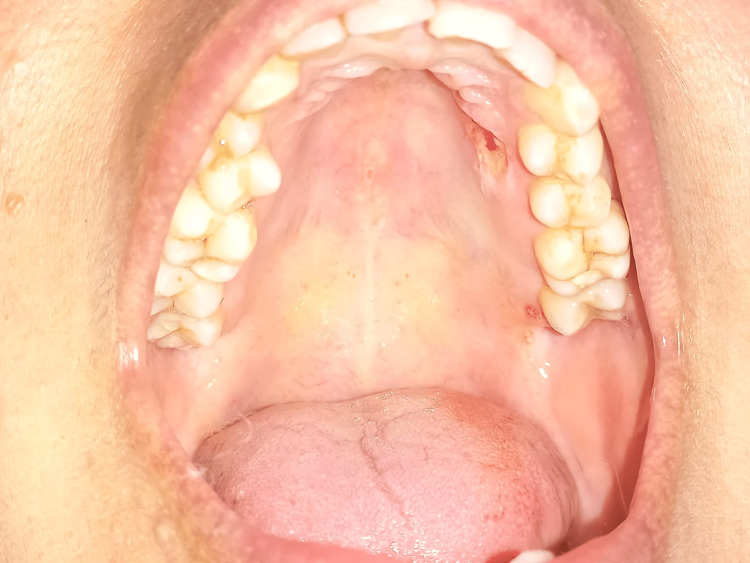
After excision

**Figure 5:**
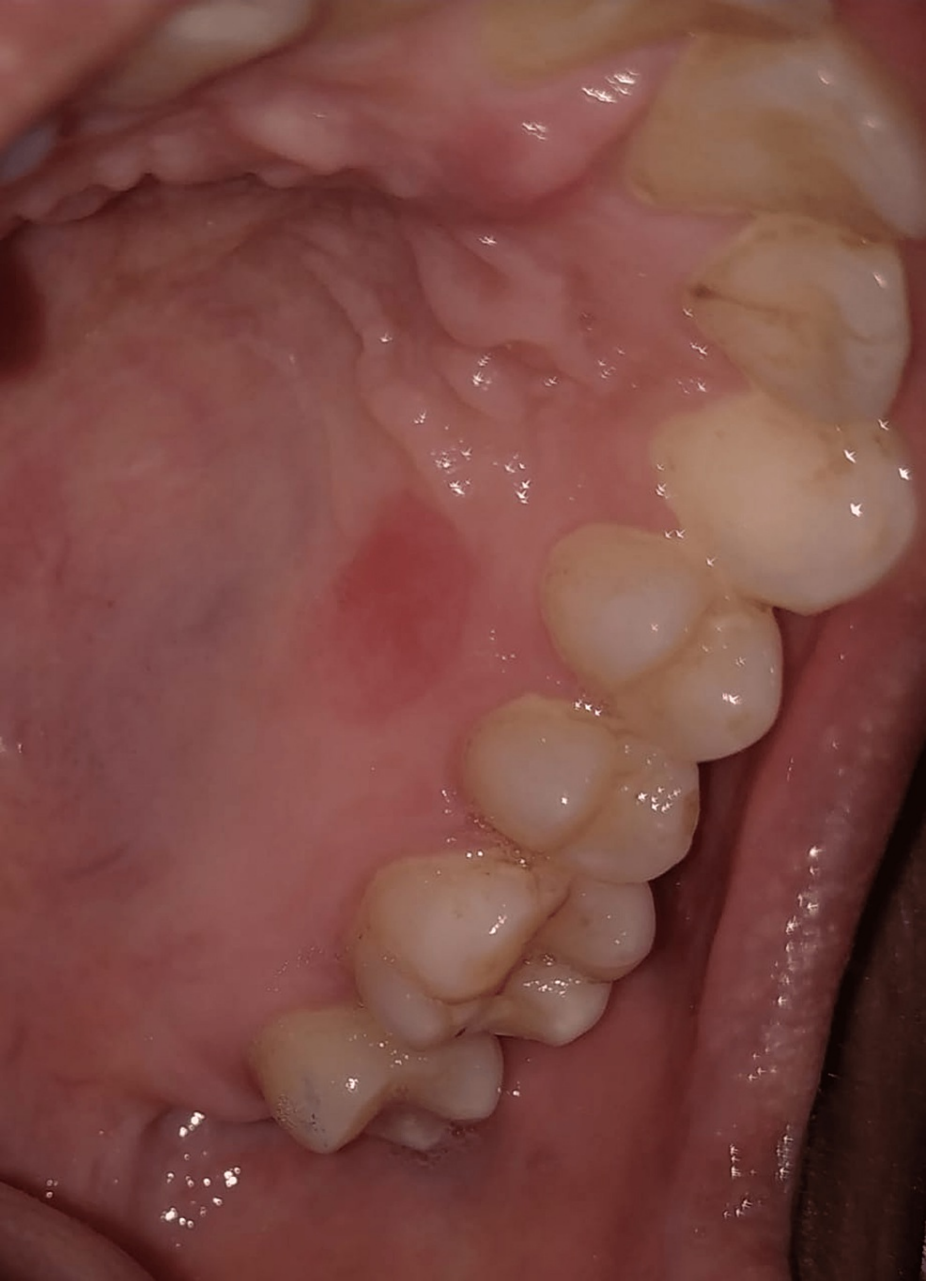
Post-operative after one week

After the removal of the lesion (Figure 3), haemostasis was achieved (Figure 4), and the tissue was sent for biopsy. Post-operative instructions and medications (Zerodol SP) twice daily for three days were given to the patient. A post-operative evaluation was done after one week (Figure 5). The lesion did not reoccur during the one-week period. A follow-up evaluation showed uneventful healing. She was advised to have regular follow-ups; however, the patient did not adhere to these instructions.

**Figure 6:**
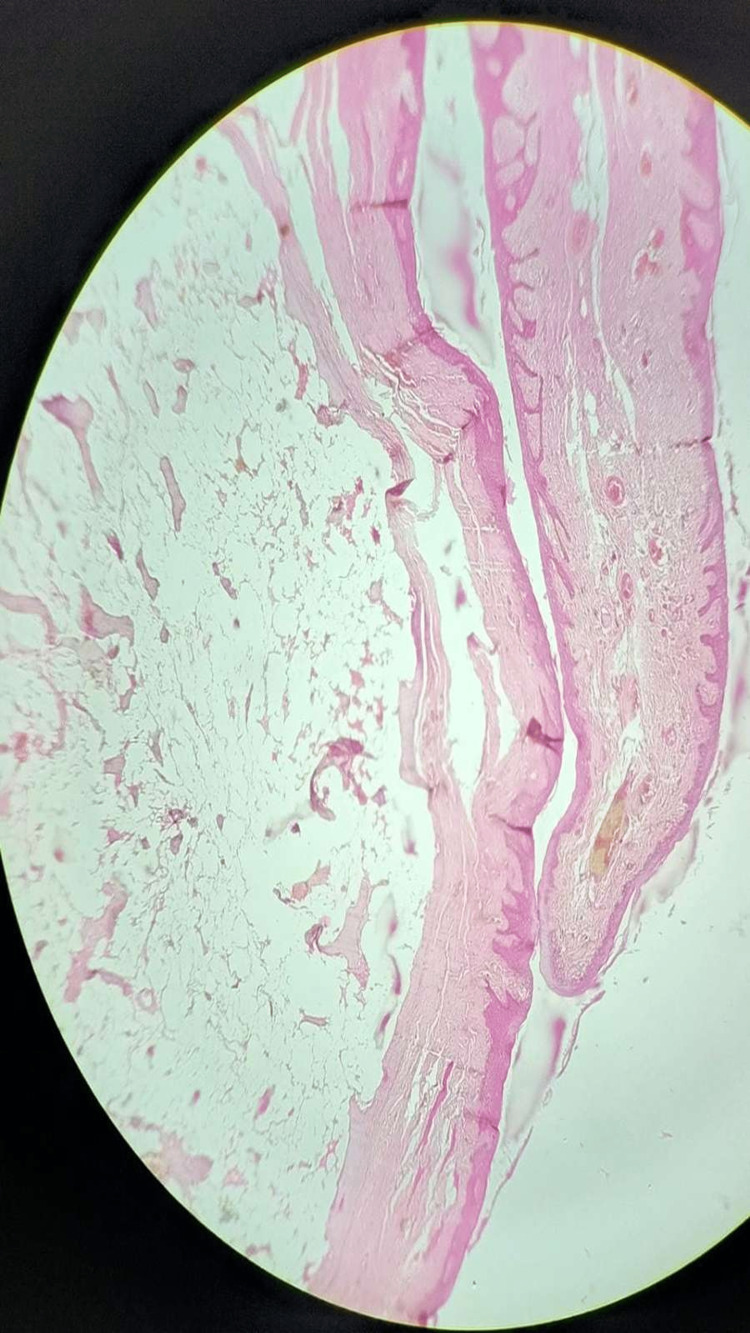
Parakeratinized stratified squamous epithelium with underlying connective tissue

Histopathological findings (Figure 6) showed a small piece of tissue with epithelium and connective tissue made of bony trabeculae and fatty marrow that was visible in the haematoxylin and eosin-stained section. The stratified squamous parakeratinized epithelium exhibits hyperplasia. The underlying connective tissue has a giant encapsulated lesion with several microscopic bony spicules in the centre and bony trabeculae around the edges. Collagen fibre bundles with inflammatory infiltrates and numerous thin-walled blood vessels with extravasated red blood cells are found in connective tissue. Overall features are suggestive of peripheral osteoma.

This report's objective is to review the literature on peripheral jaw osteomas and provide the clinical, surgical, and histological characteristics of a 42-year-old woman's maxillary peripheral osteoma that emerged in her hard palate.

## Discussion

Osteomas are benign, well-circumscribed tumours of the bone tissue that grow slowly and persistently. Osteomas are believed to form in reaction to trauma or infection, although the exact cause has not yet been determined. Osteomas are distinct lesions that are frequently found on their own. Depending on the tissue through which they originate, osteomas are classified as central, peripheral, or extra-skeletal. Peripheral osteomas (PO), central osteomas, and extra-skeletal soft tissue osteomas primarily develop from the endosteum, periosteum, and muscle tissue, respectively [4].

Depending on the location, size, and growth direction of the tumour, an osteoma may present with clinical symptoms. However, most osteomas of the maxillofacial bones are asymptomatic until they become large enough to compress or deform the nearby structures. It is indeed possible for nerve compression to create neurological symptoms [5].

Clinically, peripheral lesions resemble asymptomatic, mushroom-like masses that are unilateral and pedunculated and can cause pain, trismus (when a nerve is involved), restricted mandibular movement, malocclusion, oedema, and facial asymmetry [6].

Though the precise aetiology and pathogenesis of peripheral osteoma are yet unknown, endocrine, inflammatory, hereditary, and traumatic reasons have all been proposed as potential suspects. The majority of PO cases seem to advance slowly and show few noticeable symptoms. The finding of the PO is frequently an unintended finding. However, depending on the position and size of the tumour, some people may experience exophthalmos, headaches, facial deformities, or mandibular deviation on opening [7].

A well-defined rounded or oval radiopaque mass with a sessile or pediculated base adhering to the bone tissue is how the lesion appears on radiographs. The diagnosis is greatly aided by imaging tests like computed tomography, ultrasonography, and panoramic radiography. Computed tomography has been demonstrated in studies to be the best imaging technique to pinpoint the precise location and size of the lesion since it is adaptable, generates detailed images of bone malignancies, and has high specificity and sensitivity for abnormalities [8].

In its most recent categorization of disorders, the World Health Organization describes osteoma as a benign osseous tumour that is readily distinguishable from exostoses and tori, which are hamartomas, an approach that many authors have followed [9].

Gardner's syndrome, a phenotypic variation of familial adenomatous polyposis coli, is characterised by several osteomas. It is characterised by germline mutations in the adenomatous polyposis coli gene on the 5q chromosomal region and is inherited as an autosomal-dominant type. Other symptoms include adenomatous intestinal polyps, which have a high risk of developing into cancer, cutaneous epidermal cysts, odontomas, and extra or impacted teeth. Gardner's syndrome manifests early in the maxillofacial region as osteomas; thus, dentists and oral surgeons should refer patients for additional medical evaluation because the prognosis depends on early identification [10].

According to histology, the tumours may have cancellous or compact origins. Osteoblast-like cells that do not exhibit aberrant or malignant transformation can be seen in osteoid tissues. Numerous inflammatory or malignant lesions, including chondroma, osteochondroma, Paget's disease, Pindborg tumour, odontoma, and chronic localised sclerotic osteomyelitis, must be taken into account while determining the lesion's differential diagnosis. Peripheral osteomas are treated by completely removing the lesion surgically. Recurrence is exceedingly uncommon, and there have been no reports of malignant change following surgical excision [4].

Numerous methods for treating osteomas are documented in the literature. Due to the lesion's slow growth, conservative treatment might be used, in which the patient is monitored clinically and radiographically for a fixed timeframe to look for evidence of progression.

In the presented case, excision was done using electrocautery because the lesion was overgrown and thick. There was minimal discomfort to the patient, and it provided excellent haemostasis during the surgery and a clear visual field.

Surgical intervention is not advised when the lesion does not advance. The choice to remove the lesion surgically should be supported by a thorough evaluation of the risks of the procedure, including potential harm to important anatomical structures. When severe injuries are accompanied by a functional or cosmetic disability, surgical resection is advised [8].

## Conclusions

In the jaw bones, particularly in the maxilla, peripheral osteomas are comparatively uncommon. Most lesions are asymptomatic. Lesions are significantly more common in women than in men, and they can range in size from very little to extremely large. The chance of lesions relapsing is significantly decreased by using proper surgical methods.

Dental professionals and maxillofacial surgeons should be familiar with the clinical features and differential diagnosis of peripheral osteomas of the jaws as they are rather uncommon lesions. An early diagnosis can be crucial for the patient's prognosis because multiple osteomas may be a sign of Gardner's syndrome. In asymptomatic cases, osteomas are treated conservatively, but when necessary, surgical excision is required. Significant cosmetic deformity, functional limitations or loss, rapid development, or a confirmed histopathologic diagnosis are all indications for surgical therapy.
